# Integrative analysis and experimental validation of dioxin-interacting genes reveal diagnostic and prognostic biomarkers in lung adenocarcinoma

**DOI:** 10.1007/s10238-026-02187-3

**Published:** 2026-05-26

**Authors:** Guofang Yin, Bo Li, Shiming Fan, Zhiguo Wang, Qilan Jiang, Fang He, Hongli Cao, Yuling Liang, Ying Luo, Feng Jiang, Xianming Fan

**Affiliations:** 1 Department of Respiratory and Critical Care Medicine, The Affiliated Hospital, Southwest Medical University, Luzhou, 646000 Sichuan China; 2 Inflammation & Allergic Diseases Research Unit, The Affiliated Hospital, Southwest Medical University, Luzhou, 646000 Sichuan China; 3 Department of Pulmonary Disease, Traditional Chinese Medicine Hospital of Jiang’an County, Yibin, 644200 China; 4 Department of Cardiology, The Affiliated Hospital, Southwest Medical University, Luzhou, Sichuan China

**Keywords:** Dioxins, Lung adenocarcinoma, SLC15A2, Diagnosis, Prognosis

## Abstract

Dioxins are persistent environmental pollutants and key components of the human exposome with established carcinogenic potential. As airborne toxicants, they link environmental pollution to lung adenocarcinoma (LUAD), yet their molecular mechanisms remain unclear. Dioxin-interacting genes were curated from toxicogenomic databases and intersected with LUAD differentially expressed genes to identify dioxin-related molecular signatures. Consensus clustering was performed to define LUAD subtypes with distinct clinical and transcriptomic characteristics. Weighted gene co-expression network analysis (WGCNA) was applied to identify key gene modules associated with dioxin exposure and tumor progression. Hub genes were further integrated into diagnostic and prognostic models using multiple machine-learning algorithms based on both tumor tissue and peripheral blood transcriptomic datasets. Genome–exposome interactions were explored through pathway enrichment and chemical–gene interaction analyses. Single-cell RNA sequencing data were used to characterize cell-type–specific expression patterns. In vitro functional assays were conducted to validate the biological roles of candidate genes. Both models demonstrated robust predictive performance across cohorts. SLC15A2 was consistently identified as a hub gene and showed predominant expression in lung epithelial cells based on single-cell RNA sequencing. Pan-cancer analyses revealed significant dysregulation of SLC15A2, with lower expression in LUAD associated with poorer survival. Functional experiments confirmed that SLC15A2 overexpression suppressed LUAD cell proliferation and invasion, supporting a tumor-suppressive role. This integrative exposome–genome analysis highlights dioxin-related transcriptomic dysregulation in LUAD and identifies SLC15A2 as a potential tumor suppressor and biomarker for precision stratification.

**Supplementary Information:**

The online version contains supplementary material available at 10.1007/s10238-026-02187-3.

## Background

Lung cancer remains one of the most prevalent malignancies worldwide and continues to be the leading cause of cancer-related death. Based on data from 2020, approximately 2.2 million new cases and 1.8 million deaths were attributed to lung cancer globally [1]. In China, lung cancer accounted for about 828,000 new cases and 657,000 deaths in 2016, and it has consistently ranked as the cancer with the highest incidence and mortality in 2022 [2, 3]. With demographic aging and the persistence of major risk factors such as tobacco exposure and environmental pollution, the overall incidence of lung cancer in China has shown a rising trend [1]. Among the histological subtypes, lung adenocarcinoma (LUAD) has become the dominant type both globally and domestically [4]. Recent studies indicate that by 2020, the incidence of adenocarcinoma surpassed that of squamous cell carcinoma worldwide, with the highest proportion observed in East Asian populations and among females [4]. Emerging epidemiological evidence suggests that, beyond tobacco use, long-term contact with airborne toxicants contributes substantially to LUAD development, reflecting the influence of the broader exposome on lung carcinogenesis. These epidemiological observations highlight the substantial global and national disease burden of LUAD.

Dioxins—particularly 2,3,7,8-tetrachlorodibenzo-p-dioxin (TCDD)—are among the most hazardous and persistent pollutants within the human exposome. TCDD binds with high affinity to the aryl hydrocarbon receptor (AhR), which then translocates into the nucleus and activates the canonical AhR signaling pathway. Through this process, TCDD modulates the transcription of downstream target genes, leading to a variety of cytotoxic and potentially carcinogenic effects [5, 6]. Through sustained AhR activation, dioxin exposure perturbs gene regulation, lipid metabolism, and immune signaling, thereby establishing a mechanistic bridge between environmental contamination and cellular transformation. Studies have demonstrated that exposure to TCDD can induce epigenetic alterations across the genome, including changes in DNA methylation and regulation mediated by non-coding RNAs, thereby reshaping gene expression profiles [5]. Although the precise mechanisms remain incompletely understood, TCDD is thought to promote carcinogenesis in lung tissue primarily through activation of the AhR pathway. This process induces the expression of cytochrome P450 enzymes, which in turn generate reactive metabolites and other bioactive intermediates capable of driving mutagenesis and malignant transformation [5, 6]. In summary, current evidence indicates that dioxins may contribute to lung carcinogenesis; however, studies focusing specifically on LUAD remain limited, and further investigations are needed to elucidate the underlying mechanisms. Clarifying how dioxin-related genomic and transcriptomic alterations promote tumor initiation and progression is therefore essential for advancing mechanistic understanding and preventive strategies.

With the rapid advancement of high-throughput sequencing technologies and computational biology, genomic analysis has become an indispensable tool in biomedical research. By leveraging large-scale public repositories such as The Cancer Genome Atlas (TCGA) and Gene Expression Omnibus (GEO), investigators are able to perform differential expression profiling, weighted gene co-expression network analysis (WGCNA), and diverse machine learning–based approaches to systematically identify potential diagnostic and prognostic biomarkers in tumors and non-tumor diseases [7–9]. Recent studies further support the utility of machine-learning-based biomarker discovery in respiratory diseases. A four-gene peripheral blood–derived risk score model has been developed for LUAD diagnosis, prognosis evaluation, and immunotherapy response assessment [10]. Similarly, machine learning integrated with cellular experiments and single-cell sequencing has been used to identify endoplasmic reticulum stress–related diagnostic and prognostic biomarkers in idiopathic pulmonary fibrosis [11]. Building on these advances, the present work integrates dioxin-interacting genes with large-scale transcriptomic data to define molecular subtypes of LUAD and reveal genome–exposome interactions. By combining machine-learning–based modeling with in vitro validation, this study aims to uncover reliable biomarkers and mechanistic links connecting environmental toxicant exposure to molecular alterations in lung adenocarcinoma, thereby supporting precision prevention and targeted intervention.

## Materials and methods

### Data processing

RNA sequencing data for LUAD in fragments per kilobase of transcript per million mapped reads (FPKM) format were obtained from TCGA. These values were subsequently converted into transcripts per kilobase million (TPM) to ensure comparability with microarray-derived expression profiles. In addition, LUAD-related datasets were retrieved from the GEO database, and detailed information on these datasets is provided in Supplementary Table 1. Samples lacking complete clinical annotations or not corresponding to LUAD were excluded from further analysis. Expression data were subjected to log2 transformation, and raw profiles were normalized using the “limma” R package [12] to minimize technical variation and improve comparability across datasets. Dioxin-associated genes relevant to LUAD were retrieved from the Comparative Toxicogenomics Database (CTD) using “Dioxins” as the search keyword.

### Differential expression analysis

To identify differentially expressed genes (DEGs) between lung adenocarcinoma tissues and adjacent normal controls, expression matrices were analyzed using the limma package in R. Genes with an absolute log fold change (|logFC|) greater than 2 and an adjusted P value below 0.05 were defined as significantly differentially expressed.

### Consensus clustering analysis

Consensus clustering was applied to the gene expression profiles to delineate robust molecular subgroups. This unsupervised learning approach achieves stable sample classification by repeatedly resampling both cases and features, thereby evaluating clustering consistency across multiple iterations. The analysis was conducted using the ConsensusClusterPlus package in R, with k-means as the clustering algorithm and Euclidean distance as the similarity measure. The optimal number of subtypes was determined based on the consensus cumulative distribution function (CDF) and visualization of consensus heatmaps.

### Immune cell deconvolution

Immune infiltration was inferred from bulk transcriptomes using the Cell-type Identification By Estimating Relative Subsets Of RNA Transcripts (CIBERSORT) algorithm [13], which applies ν-support vector regression to deconvolve mixed expression profiles into relative proportions of constituent leukocyte subsets. Gene expression matrices served as input together with a predefined immune signature matrix, and significance of each deconvolution was evaluated with 1,000 label permutations. Samples with permutation P values below 0.05 were retained, and the resulting cell-fraction matrix was exported for downstream analyses.

### Weighted gene co-expression network analysis (WGCNA)

WGCNA [14] was conducted to identify modules of co-expressed genes associated with molecular subtypes. The top 5,000 most variable genes were selected to reduce noise, and outlier samples were excluded after quality control. A soft-thresholding power was determined to approximate scale-free topology, and pairwise correlations were converted into adjacency and topological overlap matrices to capture gene connectivity. Hierarchical clustering followed by dynamic tree cutting identified modules of tightly correlated genes, which were subsequently merged according to eigengene similarity. Correlations between module eigengenes and sample cluster assignments were then calculated to highlight subtype-related modules and prioritize hub genes for further analysis.

### Functional enrichment analysis

To elucidate the biological roles of the identified modules and candidate genes, Gene Ontology (GO) [15] and Kyoto Encyclopedia of Genes and Genomes (KEGG) enrichment analyses [16] were performed. GO analysis was applied to characterize biological processes, cellular components, and molecular functions associated with the gene sets, while KEGG analysis was used to identify enriched signaling and metabolic pathways. The enrichment calculations were conducted with R packages such as clusterProfiler [17], using adjusted P values to control for multiple testing. Terms or pathways with a false discovery rate (FDR) below 0.05 were considered significantly enriched.

### Construction of diagnostic prediction models

To systematically identify robust diagnostic biomarkers, a machine learning–based framework integrating variable selection and predictive modeling was established. Twelve commonly used algorithms, including Lasso, Ridge regression, Elastic Net (Enet), stepwise generalized linear modeling (Stepglm), support vector machine (SVM), generalized linear model boosting (glmBoost), linear discriminant analysis (LDA), partial least squares regression with generalized linear modeling (plsRglm), random forest, gradient boosting machine (GBM), extreme gradient boosting (XGBoost), and naïve Bayes, were implemented in different combinations. Within a cross-validation framework, one algorithm was used for feature selection and another for model construction, resulting in 113 distinct model combinations. Each model was trained on the discovery cohort and subsequently validated on independent testing datasets. Predictive performance was assessed using the area under the receiver operating characteristic curve (AUC), supplemented by confusion matrix–based classification metrics and decision curve analysis (DCA). A comparative heatmap of AUC values was also generated to illustrate the relative performance of different algorithmic strategies.

### Construction of prognostic prediction models

To identify robust prognostic signatures, a machine learning framework integrating variable selection and survival modeling was implemented. A total of 101 model combinations were evaluated, involving algorithms such as CoxBoost, random survival forest, Elastic Net (Enet), stepwise Cox regression, survival support vector machine (survivalsvm), partial least squares regression for Cox models (plsRcox), gradient boosting, and Bayesian additive regression trees. Within a cross-validation framework, one algorithm was used for variable selection and another for model building, thereby allowing comprehensive evaluation of feature selection and survival prediction strategies. Each model was trained on the discovery cohort and validated on independent testing cohorts. Candidate features with insufficient selection frequency or below a minimum threshold were excluded to avoid unstable models. The prognostic performance of each model was assessed using Harrell’s concordance index (C-index), which quantifies discriminative ability in time-to-event outcomes. Average C-index values across cohorts were visualized in a heatmap to compare algorithmic strategies and highlight the most robust model combinations. To further validate the robustness and generalizability of the proposed prognostic framework, comparative analyses were performed against previously published gene signatures. Publicly available prognostic models were collected from the literature and reconstructed using the corresponding gene sets, with reported coefficients applied when available, or refitted in the training cohort when coefficients were not provided. Both the newly developed models and the external signatures were evaluated in the same training and validation cohorts using an identical analysis pipeline. Discriminative performance was assessed by C-index, and comparative results were visualized using heatmaps and forest plots across multiple cohorts.

### Somatic mutation profiling and TMB estimation

The somatic mutation data of LUAD patients were retrieved from TCGA database. To estimate the tumor mutational burden (TMB), the total number of non-synonymous mutations detected in each LUAD sample was calculated. Furthermore, the mutational landscape of LUAD driver genes was analyzed in relation to groups stratified by different immune-related scores. Driver gene alterations were identified and visualized using the maftools package [18] in R.

### Single-cell sequencing analysis

Raw unique molecular identifier (UMI) count matrices from the GSE131907 dataset were imported into R and converted into sparse matrix format to improve computational efficiency. A total of 22 pre-defined tumor and matched normal lung samples were retained. Single-cell objects were generated using Seurat [19, 20], requiring at least 200 detected genes per cell and expression in a minimum of three cells per gene. To remove low-quality or artifactual cells, multiple filters were applied: cells with fewer than 200 or more than 10,000 detected genes were excluded, as were those with mitochondrial or ribosomal transcript fractions exceeding 20%. After quality control, only high-confidence cells were retained for downstream analyses. After quality control, gene expression counts were log-normalized and highly variable genes were identified for dimensionality reduction. Principal component analysis (PCA) was first applied to reduce noise and extract major components, and the resulting low-dimensional space was further embedded using t-distributed stochastic neighbor embedding (t-SNE) for visualization. To account for potential batch effects, data were integrated with Harmony [21] before clustering. Cell populations were then identified by graph-based clustering, and cluster-specific marker genes were determined. For cell-type annotation, the SingleR package [22, 23] was used to compare cluster-level transcriptomic profiles against a curated human reference dataset. Automated predictions were subsequently cross-checked with canonical marker genes, and manual refinements were made where necessary to ensure accurate biological interpretation.

### In silico virtual knockout analysis

To investigate potential gene-regulatory perturbations at single-cell resolution, an in silico virtual-knockout analysis was performed using the scTenifoldKnk R package. scTenifoldKnk is a single-cell gene regulatory network-based framework for simulating target-gene knockout and predicting downstream regulatory effects from scRNA-seq data [24]. The method is built on the scTenifoldNet workflow, which constructs and compares single-cell gene regulatory networks using principal-component regression, tensor decomposition, and manifold alignment [25]. The processed Seurat object was used as input, and tumor-derived cells were retained for analysis. Raw count data were extracted, and the top 5,000 highly variable genes were selected using the variance-stabilizing transformation method in Seurat. Virtual knockout was then performed using scTenifoldKnk with default quality-control procedures and the following key parameters: mitochondrial-content threshold = 0.1, minimum library size = 1,000, 10 regulatory sub-networks, and 500 randomly sampled cells per network. Differentially regulated genes were obtained from the diffRegulation output, and genes with adjusted P values < 0.05 were considered significantly perturbed. Functional enrichment analysis was subsequently performed to identify biological processes and pathways affected by the virtual knockout.

### Drug sensitivity prediction and molecular docking analysis

Drug sensitivity was predicted using data from the Genomics of Drug Sensitivity in Cancer (GDSC) database. Molecular docking was conducted with AutoDock Vina v1.2.2 to evaluate dioxin–protein interactions. The molecular structure of a representative dioxin was retrieved from PubChem, while three-dimensional protein structures were obtained from the Protein Data Bank (PDB) and AlphaFold. Prior to docking, proteins were preprocessed by adding hydrogens, removing water molecules, and converting to PDBQT format.

### Cell culture

The human LUAD cell line A549 (Shanghai, China; catalog no. SCSP-503, identifier CSTR:19375.09.3101HUMSCSP503) was purchased from the Cell Bank of the Chinese Academy of Sciences. The human normal bronchial epithelial cell line BEAS-2B (Shanghai, China; catalog no. GNHu27, identifier CSTR:19375.09.3101HUMGNHu27) was obtained from the Cell Bank of the Chinese Academy of Sciences.A549 cells were infected with a recombinant adenovirus carrying the full-length human SLC15A2 coding sequence (WZ Bioscience Inc., Shandong, China), with an empty adenoviral vector as control. After infection, stable cell populations were established, and overexpression efficiency was confirmed by quantitative real-time polymerase chain reaction (qRT-PCR).

### qRT-PCR

Total RNA from A549 cells was isolated with TRIzol (Invitrogen, USA) and reverse-transcribed using ReverTra Ace RT Master Mix (TOYOBO, Japan). Quantitative real-time PCR was carried out on an ABI 7500 system with SYBR Green chemistry, and relative SLC15A2 expression was normalized to GAPDH using the 2^–ΔΔCt method.

### Western blot (WB)

Protein lysates from A549 cells (adenoviral SLC15A2 or vector control) were prepared in RIPA buffer with protease/phosphatase inhibitors and quantified by BCA. Equal amounts of protein (20–40 µg) were separated by SDS–PAGE, transferred to PVDF, blocked in 5% non-fat milk/TBST, and probed with primary antibodies against SLC15A2 and GAPDH (4 °C, overnight) followed by IRDye 800CW secondary antibodies; signals were imaged on a LI-COR Odyssey CLx and quantified by densitometry with normalization to GAPDH.

### Colony formation assay

A549 cells with or without SLC15A2 overexpression were plated at low density and cultured until visible colonies appeared. Colonies were fixed, stained with crystal violet, and quantified; relative plating efficiency was compared between groups.

### Transwell assay

Cell migration was assessed using Transwell chambers with serum as chemoattractant. After incubation, non-migrated cells were removed, while migrated cells were fixed, stained, and quantified microscopically.

### Statistical analyses

All statistical analyses were performed with R software (version 4.3.1). For comparisons among multiple groups, the Kruskal–Wallis test was applied, whereas differences between two groups were assessed using the Wilcoxon rank-sum test. In survival analyses, the optimal cutoff value was identified through the surv_cutpoint function, which selects the threshold that yields the greatest separation of survival curves. Kaplan–Meier estimates were used to visualize survival probabilities, and differences between groups were examined by the log-rank test. The relationship between risk score subgroups and somatic mutation frequencies was assessed using a chi-square test. Associations between variables were assessed using Spearman’s correlation analysis. A two-sided p-value < 0.05 was regarded as statistically significant.

## Results

### Subtype classification and prognostic relevance of dioxin-linked genes in LUAD

**Fig. 1:**
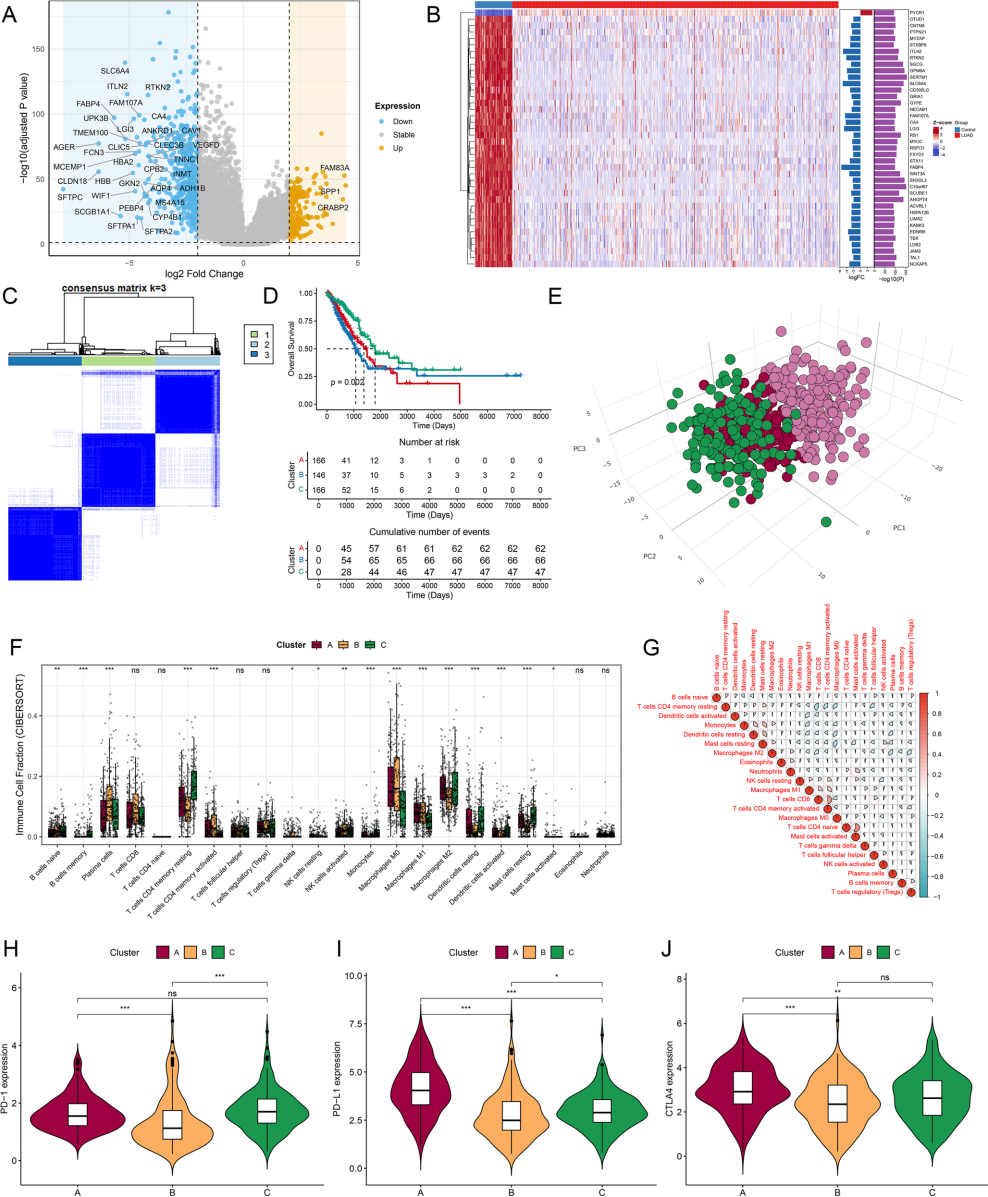
Identification of molecular subtypes based on dioxin-related gene expression patterns in LUAD. (**A**) Volcano plot illustrating differentially expressed genes (DEGs) between LUAD and normal tissues in the TCGA cohort, and (**B**) heatmap displaying their expression profiles. (**C**) Consensus clustering matrix shows the robustness of classifying LUAD samples into three distinct molecular subtypes based on intersected expression patterns of DEGs and dioxin-interacting genes. (**D**) Kaplan–Meier survival curves indicate significant differences in prognosis among the three subtypes, while (**E**) principal component analysis (PCA) demonstrates clear separation between them. (**F**) The distribution of immune cell infiltration significantly varies across the three subtypes, and (**G**) correlation analysis highlights the relationships among immune cell populations within LUAD samples. (**H–J**) Differential expression of key immune checkpoint molecules, including PD-1, PD-L1, and CTLA-4, further underscores the immunological heterogeneity between molecular subtypes

The basic information of the datasets used in this study is provided in Supplementary Table 1. As illustrated in Fig. 1A and B, differential expression analysis between LUAD tissues and adjacent normal samples in the TCGA-LUAD cohort identified 550 DEGs. These DEGs were then intersected with dioxin-related genes from the CTD database, filtered for those with a Reference Count greater than 5, resulting in 229 overlapping genes. Consensus clustering based on these 229 genes identified three distinct molecular subtypes (Fig. 1C). Survival analysis demonstrated significant prognostic differences among the clusters, with Cluster C exhibiting the most favorable outcome, Cluster A showing intermediate prognosis, and Cluster B associated with the poorest survival (Fig. 1D). Principal component analysis further confirmed a clear separation among the three subgroups (Fig. 1E).

### Immune landscape and checkpoint expression differences among LUAD clusters

Subsequent immune infiltration analysis revealed significant differences in the proportions of several immune cell subsets among the three clusters (Fig. 1F). These included naïve B cells, memory B cells, plasma cells, resting memory CD4⁺ T cells, activated memory CD4⁺ T cells, γδ T cells, resting NK cells, activated NK cells, monocytes, M0 macrophages, M1 macrophages, M2 macrophages, resting dendritic cells, activated dendritic cells, resting mast cells, and activated mast cells. Correlation analysis further indicated notable associations among certain immune cell types (Fig. 1G); for instance, CD8⁺ T cells showed a significant positive correlation with activated memory CD4⁺ T cells, while CD4⁺ naïve T cells correlated positively with activated mast cells, and CD8⁺ T cells were negatively correlated with resting memory CD4⁺ T cells. In addition, expression levels of immune checkpoint molecules differed across clusters (Fig. 1H-J). For example, PD-1 expression was comparable between Clusters A and C, and CTLA-4 expression did not differ significantly between Clusters B and C. In contrast, PD-1, PD-L1, and CTLA-4 exhibited significant expression differences among the remaining cluster comparisons.

### WGCNA Identifies Subtype-Associated Gene Modules in LUAD

**Fig. 2:**
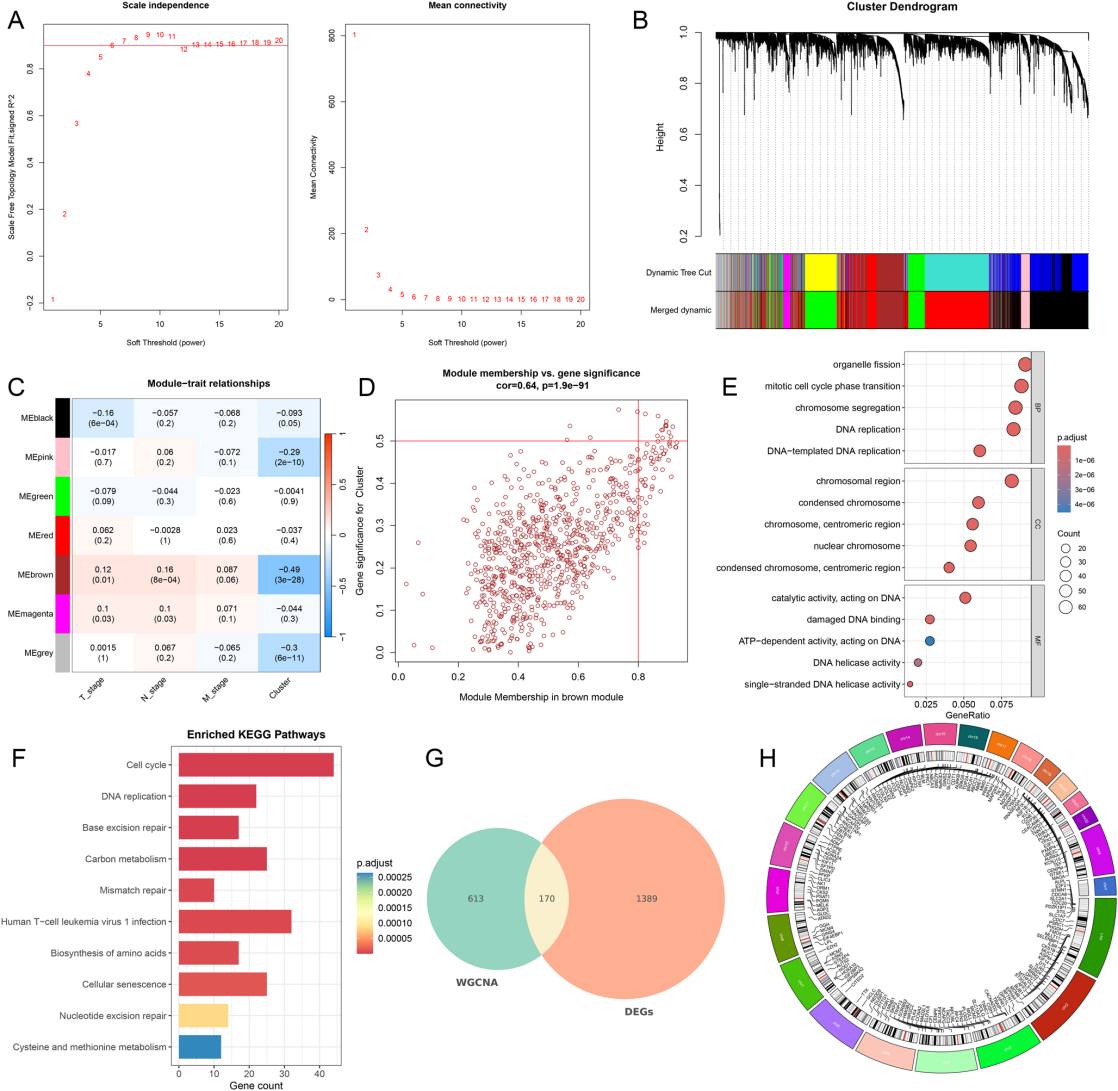
Identification of key co-expression modules and hub genes associated with molecular subtypes. (**A**) Determination of the optimal soft-thresholding power (β) based on the scale-free topology criterion (left) and the corresponding mean connectivity (right), ensuring a biologically meaningful network structure. (**B**) Hierarchical clustering dendrogram of genes, with co-expression modules identified by dynamic tree cutting and subsequently merged based on similarity, where each color represents a distinct functional module. (**C**) Heatmap depicting correlations between module eigengenes and clinical traits (T/N/M stage and cluster classification), with the brown module exhibiting the strongest negative correlation with molecular subtypes. (**D**) Scatter plots illustrating the relationship between gene significance and module membership within the brown module, highlighting genes that are highly relevant to both the module structure and clinical features. (**E**) Gene Ontology (GO) enrichment analysis showing the biological processes significantly associated with genes in the brown module, and (**F**) KEGG pathway analysis revealing key signaling pathways involved. (**G**) Venn diagram displaying the intersection between DEGs across molecular subtypes and genes from the brown module, identifying a total of 170 candidate hub genes. (**H**) Chromosomal landscape of these 170 hub genes, showing their distribution across different chromosomes

WGCNA was performed using the TCGA-LUAD transcriptome dataset to explore gene modules associated with the three LUAD clusters identified previously. Quality control indicated that the dataset contained no obvious missing values or outliers. The soft-thresholding power (β) was set to 6, as this was the lowest value at which the scale-free topology index reached a stable plateau (Fig. 2A). Based on this parameter, a topological overlap matrix (TOM) was generated and used for module detection. After merging modules with highly similar eigengene profiles, seven distinct gene modules were obtained (Fig. 2B). Among them, the brown module exhibited the strongest negative correlation with the LUAD clusters (*r* = − 0.49, *p* = 3 × 10⁻²⁸; Fig. 2C). To further validate the biological significance of this module, the relationship between module membership (MM) and gene significance (GS) was examined. A strong positive association was observed (cor = 0.64, *p* = 1.9 × 10⁻⁹¹; Fig. 2D), indicating that hub genes within the brown module are highly relevant to LUAD subtype features and thus provide a solid basis for downstream functional analyses.

### Functional enrichment and hub gene identification

Functional enrichment analysis of the brown module indicated that its genes were predominantly involved in cell cycle regulation, chromosome organization, and DNA replication, with molecular functions enriched in DNA helicase and DNA-binding activities (Fig. 2E). KEGG pathway analysis further emphasized the enrichment of cell cycle, DNA replication, and several DNA repair pathways (Fig. 2F). Differential expression analysis across the three LUAD clusters, using thresholds of |logFC| > 1 and adjusted p-value < 0.05, identified 1,559 DEGs. Intersecting these DEGs with the genes from the brown module yielded 170 hub genes (Fig. 2G; Supplementary Table 2), which were distributed across all chromosomes except chromosome 13 and the Y chromosome (Fig. 2H).

### Diagnostic model construction and clinical utility assessment

**Fig. 3:**
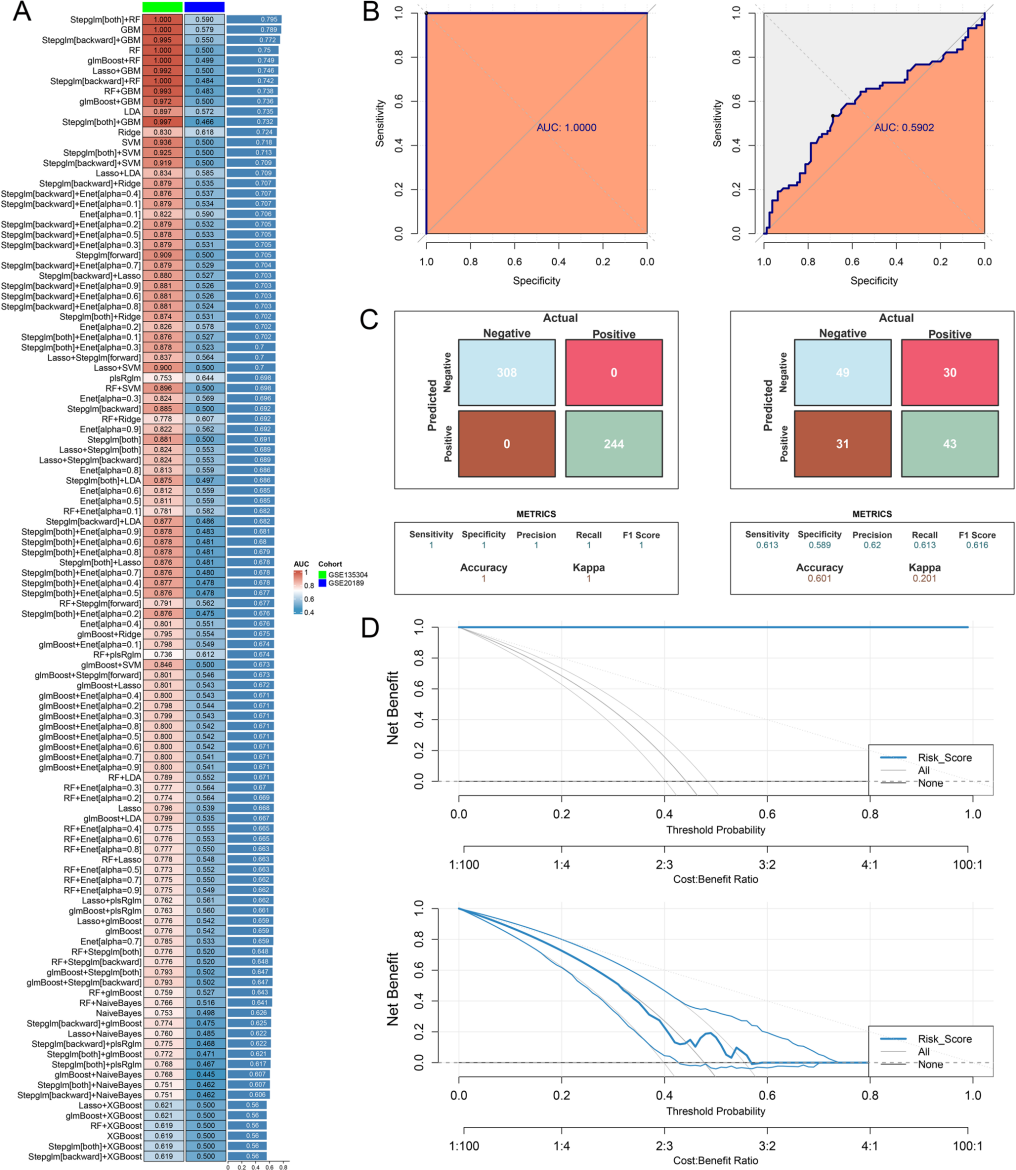
Construction and evaluation of a diagnostic model for LUAD using multiple machine learning algorithms. (**A**) Development of a LUAD diagnostic model integrating multiple machine learning algorithms based on peripheral blood transcriptomic data. (**B**) Receiver operating characteristic (ROC) curves and corresponding area under the curve (AUC) values demonstrating the diagnostic performance of the model in two independent validation cohorts: GSE135304 (left) and GSE20189 (right). (**C**) Confusion matrices illustrating the classification accuracy of the diagnostic model in the GSE135304 (left) and GSE20189 (right) cohorts. (**D**) Decision curve analysis (DCA) evaluating the clinical utility and net benefit of the diagnostic model in GSE135304 (top) and GSE20189 (bottom)

Because histopathological biopsy is invasive and unsuitable for repeated assessments, peripheral whole blood was employed as a non-invasive and easily accessible sample source. Blood-derived molecular signatures can reflect tumor-associated alterations and thereby provide a practical foundation for diagnostic model development in LUAD. Two blood-based transcriptomic datasets were included in this study, with GSE135304 used as the training cohort and GSE20189 as the validation cohort. As illustrated in Fig. 3A, multiple machine learning strategies were compared, among which the Stepglm[both] + RF model (Supplementary Table 3) yielded the highest average AUC (0.795). ROC analysis demonstrated that the model reached an AUC of 1.0 in the training dataset (GSE135304) and 0.59 in the validation dataset (GSE20189) (Fig. 3B). Confusion matrix analysis further indicated that the model achieved near-perfect classification in the training cohort but only moderate performance in the external validation set, highlighting variability in predictive accuracy across datasets (Fig. 3C). To assess potential clinical utility, decision curve analysis (DCA) was conducted. In GSE135304, the risk score consistently delivered a greater net benefit than either the “treat all” or “treat none” strategies across a broad range of threshold probabilities, suggesting strong clinical applicability. In GSE20189, the model also provided measurable net benefit, particularly within low-to-moderate probability thresholds, thereby supporting its robustness and potential value in clinical decision-making (Fig. 3D).

### Machine learning–driven construction and validation of prognostic models

**Fig. 4:**
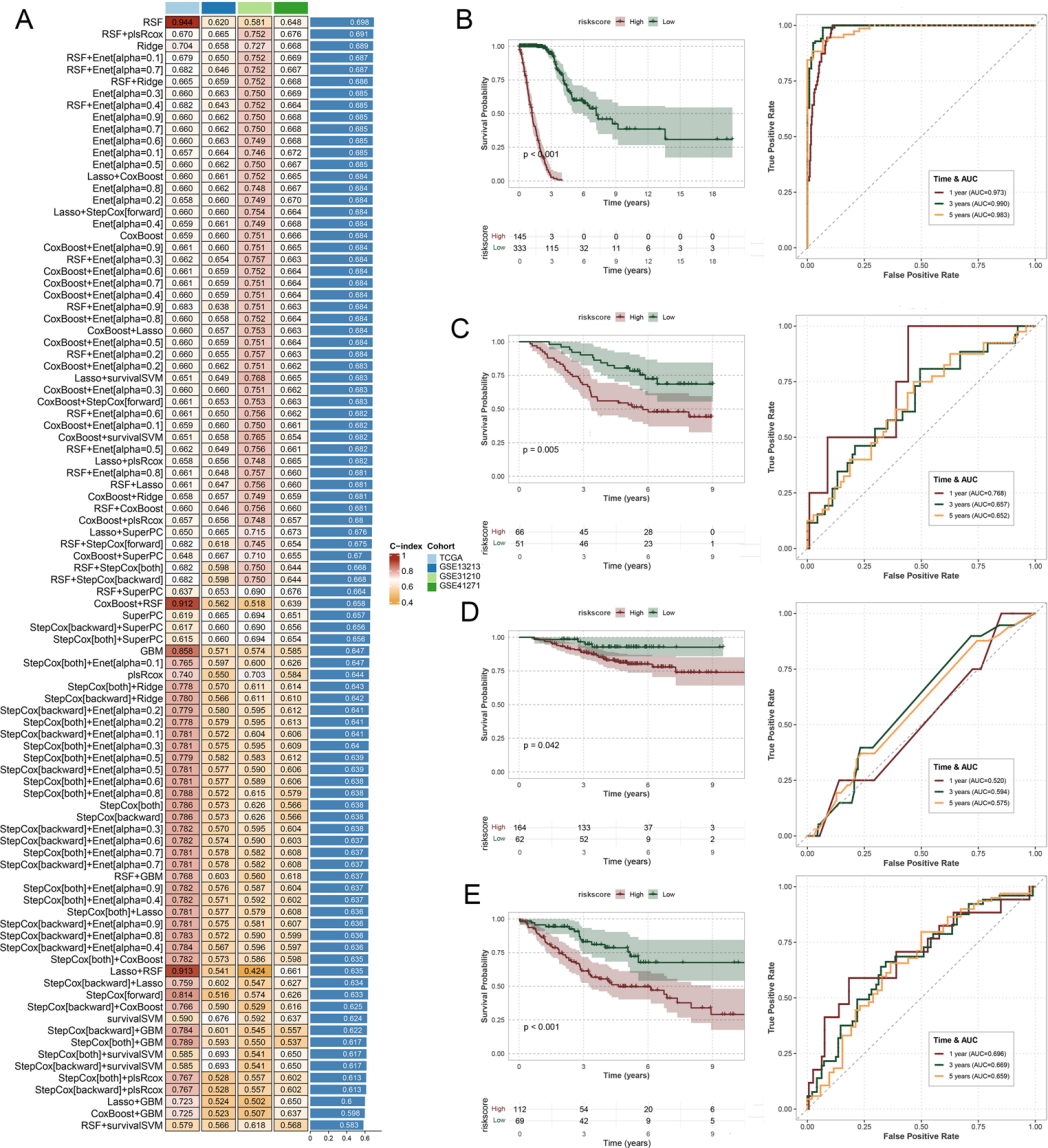
Construction and validation of a prognostic model for LUAD based on multiple machine learning algorithms. (**A**) Development of a prognostic model for LUAD using an integrated machine learning framework based on key hub genes. Kaplan–Meier survival curves and time-dependent receiver operating characteristic (ROC) analyses were employed to assess the predictive performance of the model in multiple independent cohorts: (**B**) TCGA cohort, (**C**) GSE13213 cohort, (**D**) GSE31210 cohort, and (**E**) GSE41271 cohort

Using the 170 hub genes, prognostic models were systematically constructed through multiple machine learning algorithms, with TCGA-LUAD as the training cohort and GSE13213, GSE31210, and GSE41271 as external validation datasets (Fig. 4A). Among the tested strategies, the Random Survival Forest (RSF) model achieved the highest mean C-index (0.698) and was selected as the final prognostic model (Supplementary Table 4). Kaplan–Meier survival analysis demonstrated that patients stratified into the high-risk group by the RSF-derived risk score had significantly worse overall survival compared with those in the low-risk group across training and validation cohorts (Fig. 4B). Time-dependent ROC analysis further confirmed the robust predictive capacity of the model, with the risk score showing favorable AUC values at 1-, 3-, and 5-year intervals (Fig. 4C). Importantly, multivariate Cox regression revealed that, except in the GSE31210 dataset, the risk score consistently served as an independent prognostic indicator after adjustment for conventional clinical variables (Fig. 4D). To benchmark the predictive utility of the RSF-derived signature, its performance was compared against a series of previously published LUAD prognostic models across TCGA (training set) and three independent GEO validation cohorts (GSE13213, GSE31210, and GSE41271) ((Supplementary Fig. 1). In the TCGA dataset, the RSF model demonstrated the highest C-index among all tested signatures, highlighting its superior prognostic accuracy. Although variability was observed across external cohorts, the RSF model consistently ranked among the top-performing approaches and achieved stable predictive capacity relative to most reported models.

### Immune cell infiltration and somatic variant distribution in LUAD subgroups

**Fig. 5:**
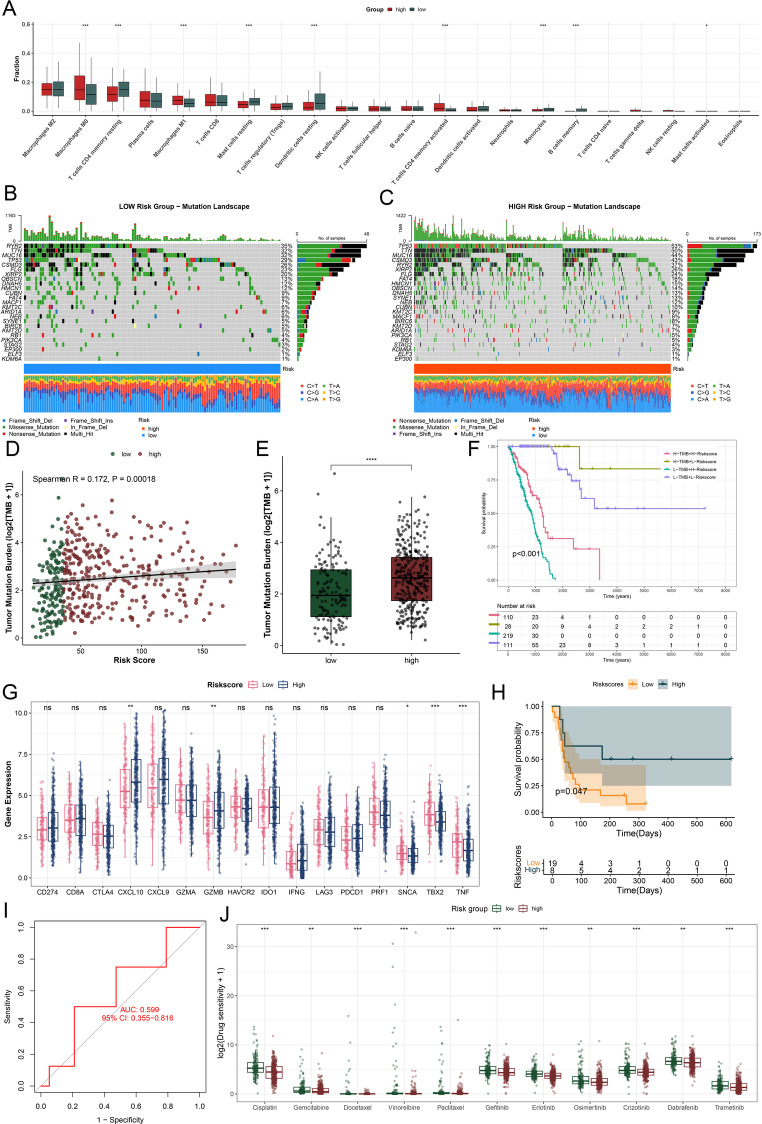
Comprehensive immunogenomic and therapeutic landscape associated with risk score stratification in LUAD. (**A**) Comparison of immune cell infiltration profiles between high- and low-risk groups. (B, C) OncoPrint plots illustrating the mutational landscapes of patients with low (**B**) and high (**C**) risk scores, with each column representing an individual patient. (**D**) Correlation analysis between tumor mutational burden (TMB) and risk scores. (**E**) Differences in TMB levels between the two risk groups. (**F**) Kaplan–Meier survival curves for patients in the TCGA-LUAD cohort stratified by both TMB status and risk score. (**G**) Expression patterns of immune checkpoint-related genes and immune activation-associated genes in high- and low-risk groups. (**H**) Kaplan–Meier survival curves for patients in the GSE135222 cohort stratified by risk score. (**I**) Receiver operating characteristic (ROC) curves evaluating the predictive performance of risk scores for immunotherapy response in the GSE135222 cohort. (**J**) Comparative analysis of the sensitivity of different risk subgroups to various antitumor agents

Immune cell infiltration profiles were compared between the high- and low-risk groups in the TCGA-LUAD cohort (Fig. 5A). Significant differences were observed in the abundance of T cells CD4 memory activated, T cells regulatory (Tregs) and some immune cells. T cells CD4 memory activated were more prevalent in the high-risk group, while Tregs exhibited higher proportions in the low-risk group, suggesting that these immune cell subsets may be associated with prognosis in LUAD. In addition, somatic mutations in LUAD driver genes were analyzed between the high- and low-risk subgroups. The maftools package was used to identify the top 25 driver genes with the highest mutation frequencies (Fig. 5B and C). Analysis of the mutation data from the TCGA cohort showed that the alteration frequencies of TP53, TTN, CSMD3, COL22A1, SI, HCN1, and LCT were significantly different between the two risk groups (Supplementary Table 5).

### TMB and immune activity signatures in LUAD subgroups

Recent studies have suggested that TMB may be associated with cancer prognosis [26, 27]. In this study, differences in TMB were analyzed between high- and low-risk LUAD patients. A weak positive correlation was observed between TMB and the risk score (Fig. 5D), with high-risk patients exhibiting significantly higher TMB than low-risk patients (Fig. 5E). Stratified survival analysis demonstrated that TMB status did not interfere with the prognostic accuracy of the risk score-based model, as significant survival differences were observed across both high and low TMB subgroups (Fig. 5F). Additionally, immune activity-related signatures [28] were compared between risk subgroups. CXCL10 and GZMB were highly expressed in the high-risk group, whereas SNCA, TBX2, and TNF showed higher expression in the low-risk group (Fig. 5G), suggesting that immune microenvironment differences contribute to the observed prognostic outcomes. The GSE135222 dataset, which includes data from non-small cell lung carcinoma (NSCLC) patients treated with anti-PD-1/PD-L1 therapies, further confirmed that high-risk patients had worse survival outcomes compared to low-risk patients, with the risk score also showing promise in predicting immune therapy responses (Fig. 5H and I). Moreover, drug sensitivity profiles from the Genomics of Drug Sensitivity in Cancer database revealed that the low-risk group exhibited significantly higher sensitivity to various chemotherapy agents and targeted therapies, including Cisplatin, Gemcitabine, Docetaxel, Vinorelbine, Paclitaxel, and Gefitinib (Fig. 5J). These findings highlight the potential clinical utility of the risk score in both prognostic assessment and treatment decision-making in LUAD.

### Single-cell transcriptomics reveals cellular heterogeneity and lineage-specific expression of SLC15A2 in LUAD

**Fig. 6:**
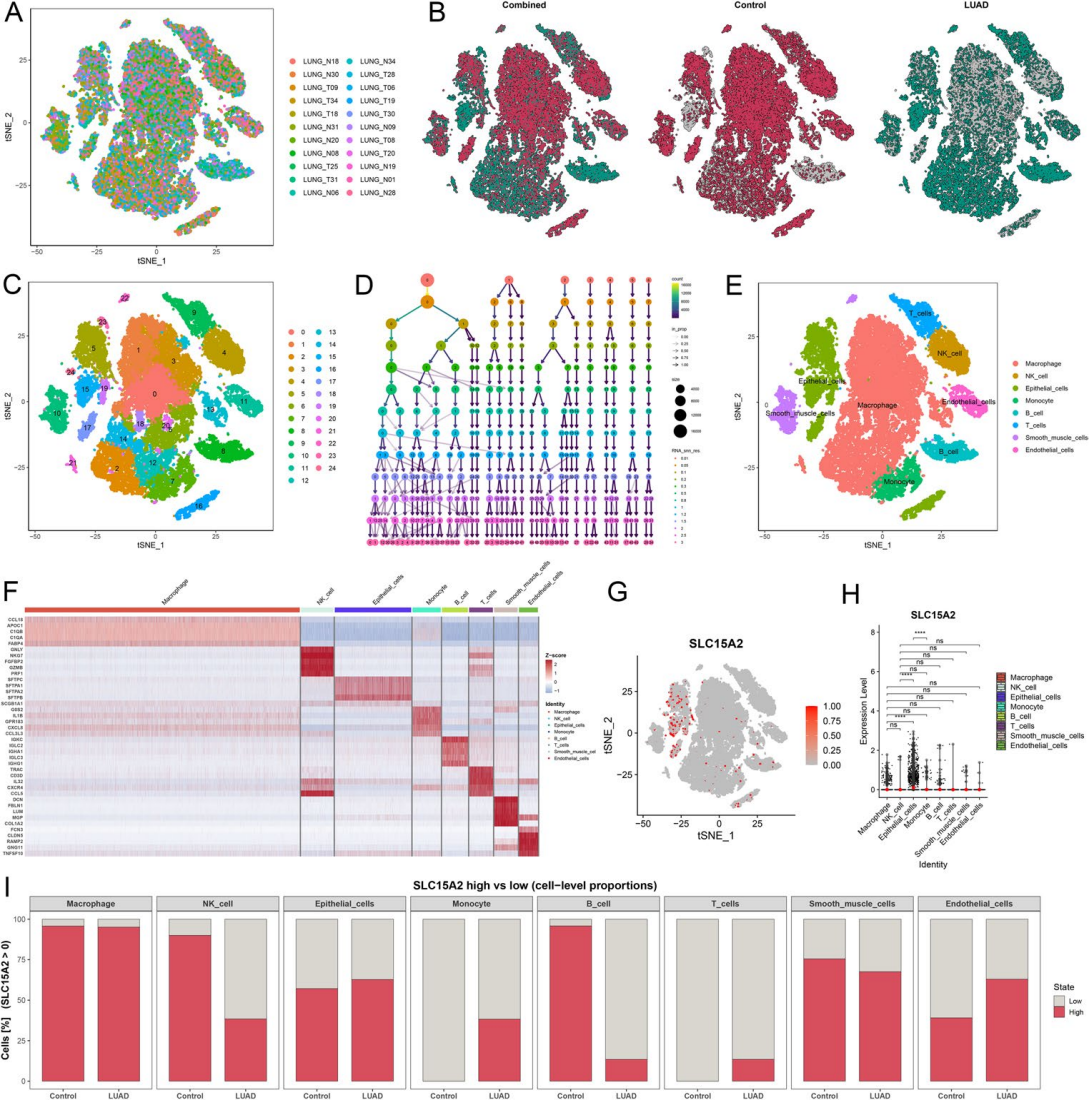
Single-cell transcriptomic landscape and SLC15A2 expression patterns in LUAD. (**A**) t-distributed stochastic neighbor embedding (t-SNE) visualization of the 22 analyzed samples. (**B**) Comparative t-SNE plots highlighting the transcriptional differences between LUAD and control groups. (**C**) t-SNE–based clustering analysis identifying 25 distinct cellular populations. (**D**) Clustering strategy of the single-cell data using 17 principal components (PCs) and a resolution of 1.2. (**E**) Classification and annotation of eight major cell types. (**F**) Heatmap showing the top five marker genes across the identified cell types. (**G**) Distribution of SLC15A2 expression among different cell populations. (**H**) Differential expression analysis of SLC15A2 across major cell types. (**I**) Proportional comparison of cells with varying SLC15A2 expression levels between LUAD and control samples

After stringent quality control steps, including the removal of doublets, apoptotic cells, and empty droplets, a high-confidence single-cell transcriptomic dataset was generated (Supplementary Fig. 2). To correct for sample-derived batch effects, the Harmony algorithm was applied, which substantially improved the integration of cells across LUAD and control lung tissues (Fig. 6A, B). Unsupervised clustering identified 25 transcriptional clusters (Fig. 6C), and clustree analysis further confirmed the stability and hierarchical structure of these clusters at varying resolutions (Fig. 6D). Cell type identities were assigned using the SingleR package with the Human Primary Cell Atlas as a reference, enabling the classification of major immune and stromal populations, including macrophages, T cells, B cells, NK cells, epithelial cells, monocytes, endothelial cells, and smooth muscle cells (Fig. 6E). Macrophages represented the dominant cell population, while epithelial and immune subsets were clearly delineated, highlighting the cellular diversity and immune–stromal interactions within the LUAD microenvironment. Marker gene expression profiles supported the reliability of these annotations (Fig. 6F). Among the hub genes, SLC15A2 was selected for further analysis because it was consistently implicated in both the diagnostic and prognostic models. Visualization on the t-SNE map revealed that SLC15A2 expression was predominantly enriched in epithelial cells, with only limited expression across immune and stromal lineages (Fig. 6G). Quantitative analysis demonstrated significantly higher SLC15A2 expression in epithelial cells compared with other cell types (Fig. 6H), suggesting its potential as a biomarker relevant to LUAD progression and clinical outcome. Furthermore, stratification of cells into SLC15A2-high and SLC15A2-low groups (based on detectable versus undetectable expression) revealed distinct distribution patterns between LUAD and control samples (Fig. 6I). In control lung tissues, SLC15A2-high cells were mainly enriched within NK and B cells, whereas in LUAD samples, SLC15A2-high cells were more frequently observed in T cells and endothelial populations, indicating disease-associated shifts in SLC15A2 expression across immune and stromal compartments.

### In silico virtual knockout analysis of SLC15A2

To further explore the potential regulatory consequences of SLC15A2 loss, we performed an in silico virtual-knockout analysis using scTenifoldKnk based on the single-cell RNA-seq dataset. Tumor-derived cells were retained for this analysis, whereas control cells were excluded. After SLC15A2 virtual knockout, 2.6% of the analyzed genes were significantly perturbed (Supplementary Fig. 3A). The top differentially regulated genes included ALCAM, EPCAM, ELF3, PKP3, PSMB5, LMO7, HIST1H2BD, PAWR, PRPF19, and STC1 (Supplementary Fig. 3B, C). Functional enrichment analysis showed that the significantly perturbed genes were mainly associated with cell–matrix adhesion, cell–substrate adhesion, integrin-mediated signaling, extracellular matrix organization, collagen fibril organization, regulation of epithelial-cell proliferation, and regulation of cell migration (Supplementary Fig. 3D). Pathway enrichment analysis further highlighted focal adhesion, integrin signaling, ECM–receptor interaction, PI3K–Akt signaling pathway, EGFR tyrosine kinase inhibitor resistance, xenobiotic metabolism by cytochrome P450, and chemical carcinogenesis-related pathways (Supplementary Fig. 3E). These findings suggest that SLC15A2 loss may affect tumor-cell adhesion, extracellular-matrix remodeling, migration-related programs, and toxicant-response pathways in LUAD.

### SLC15A2 as a potential tumor suppressor in LUAD

**Fig. 7:**
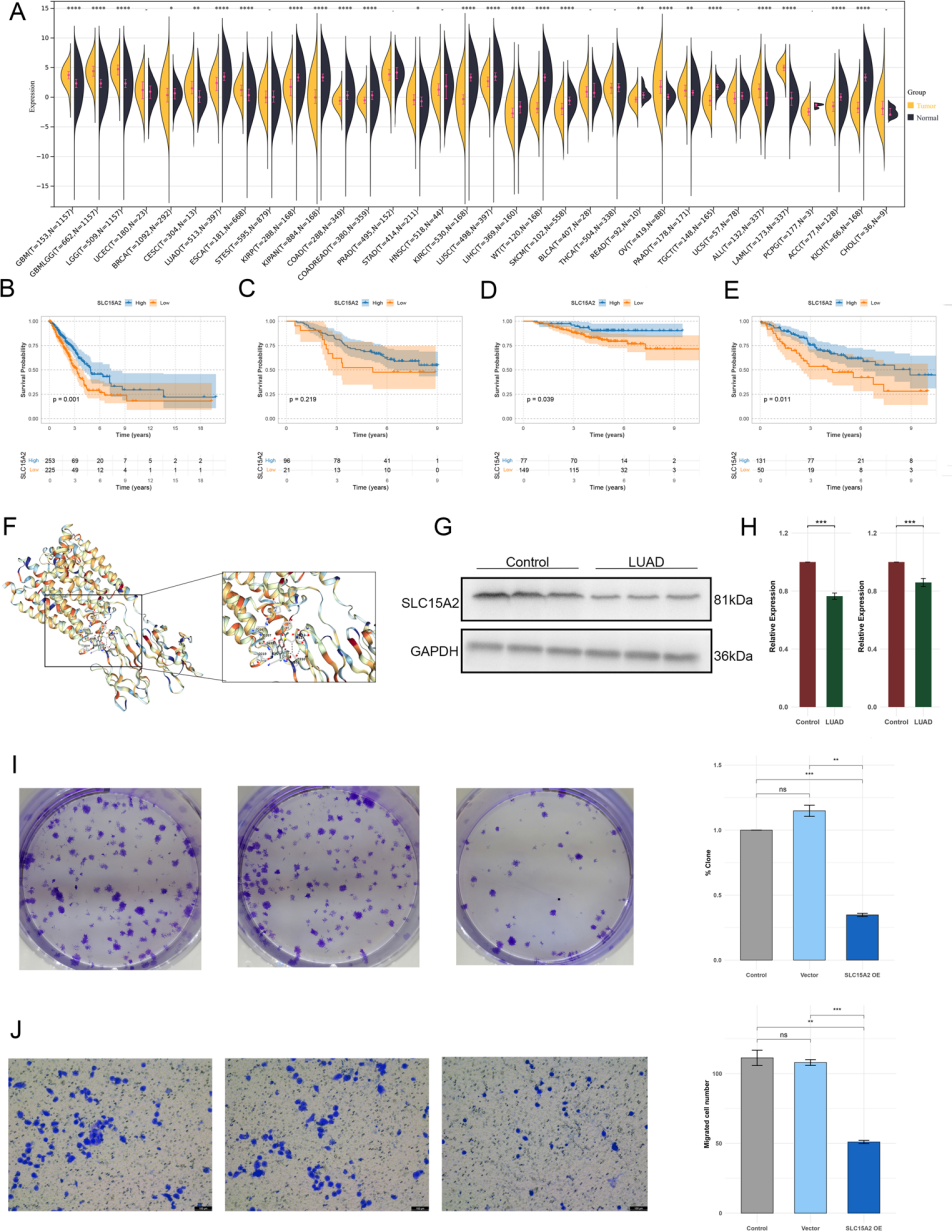
Functional characterization of SLC15A2 expression, clinical relevance, and biological effects in LUAD. (**A**) Pan-cancer analysis demonstrating significantly dysregulated expression of SLC15A2 across multiple cancer types. (**B–E**) Kaplan–Meier survival analyses showing the prognostic impact of SLC15A2 expression levels in the TCGA (**B**), GSE13213 (**C**), GSE31210 (**D**), and GSE41271 (**E**) cohorts. (**F**) Molecular docking analysis illustrating the predicted binding of SLC15A2 protein to dioxin, highlighting the complex with the lowest binding energy. (**G**) Western blot results comparing SLC15A2 protein expression between LUAD cells and normal lung epithelial cells. (**H**) Quantitative comparison of SLC15A2 expression levels between LUAD and normal epithelial cells by qRT-PCR and western blot analysis. (**I**) Colony formation assay showing the effects of SLC15A2 overexpression on LUAD cell proliferation, with representative images of the control, empty vector, and SLC15A2-overexpressing groups from left to right. (**J**) Transwell invasion assay demonstrating the impact of SLC15A2 overexpression on LUAD cell invasive capacity, with representative images of the control, empty vector, and SLC15A2-overexpressing groups from left to right

Pan-cancer analysis demonstrated that SLC15A2 expression differed significantly between tumors and adjacent normal tissues across multiple cancer types (Fig. 7A), with notably reduced expression observed in LUAD. Across the four prognostic datasets assessed, elevated SLC15A2 expression was generally associated with improved survival in LUAD patients, with the exception of the GSE13213 cohort (Fig. 7B). To further explore potential mechanistic links, molecular docking simulations were performed using AutoDock Vina. The three-dimensional structures of SLC15A2 proteins were retrieved from PubChem, and docking was carried out to calculate binding affinities. The results indicated strong electrostatic interactions and high binding stability between dioxins and SLC15A2 proteins, suggesting the formation of stable ligand–protein complexes (Fig. 7C). Experimental validation further supported these findings: qRT-PCR and western blot analyses confirmed that SLC15A2 expression was markedly lower in A549 cells than in normal lung epithelial cells (Fig. 7D, E). Functionally, colony formation assays showed that forced overexpression of SLC15A2 significantly inhibited the proliferative capacity of A549 cells (Fig. 7F), while transwell assays revealed that SLC15A2 overexpression suppressed their invasive ability (Fig. 7G).

## Discussion

In this study, dioxin-interacting genes were utilized to stratify lung adenocarcinoma (LUAD) into distinct molecular subtypes through unsupervised clustering, each characterized by unique transcriptional and prognostic landscapes. WGCNA further uncovered key co-regulated gene modules and pivotal hub genes underlying these subtype-specific differences. Based on peripheral blood transcriptomic profiles, a diagnostic model was constructed, while hub genes were also used to establish a prognostic model whose risk score not only predicted survival but also correlated with immunotherapy response and differential sensitivity to several antitumor agents. Among the identified candidates, SLC15A2 emerged as a gene present in both diagnostic and prognostic signatures. Single-cell RNA sequencing revealed its predominant expression in epithelial cells, whereas pan-cancer analysis indicated widespread dysregulation across malignancies, with notably reduced levels in LUAD. Importantly, LUAD patients with lower SLC15A2 expression had poorer prognosis. Experimental validation by qRT-PCR and western blot confirmed downregulation of SLC15A2 in LUAD cells compared with normal lung epithelial cells. Functional assays demonstrated that enforced SLC15A2 overexpression suppressed LUAD cell proliferation and invasion, underscoring its potential role as a tumor suppressor and biomarker for diagnosis and prognosis.

Epidemiological studies have long raised concerns about the potential carcinogenic effects of dioxins, particularly TCDD, yet the relationship with lung cancer remains inconclusive. While some meta-analyses have suggested a possible association between dioxin exposure and increased cancer risk or mortality, especially for certain hematological malignancies, findings across solid tumors such as lung cancer have been inconsistent [29, 30]. These discrepancies underscore the complexity of dioxin-related carcinogenesis and suggest that indirect mechanisms, such as immune modulation, metabolic disruption, or interaction with specific gene networks, may be involved. In this context, focusing on dioxin-interacting genes provides a rational approach to explore how environmentally induced molecular alterations could shape tumor biology. By integrating these genes into clustering, prognostic modeling, and functional analyses, it becomes possible to uncover novel molecular subtypes and biomarkers with both mechanistic and translational relevance in LUAD.

Compared with previous blood-derived gene expression signatures, our eight-gene diagnostic model demonstrates clear advantages in simplicity, biological relevance, and validation. The model was developed using the GSE135304 cohort and independently validated in the GSE20189 dataset, confirming its strong generalisability across different blood-based platforms. Earlier models often included large gene panels. For instance, one study employed a random forest algorithm to construct an 87-gene classifier from peripheral blood [31], while another used logistic regression to develop a 12-gene signature from the GSE20189 dataset [32]. However, these models lacked external validation, biological interpretability, or were built from limited sample populations. In contrast, our approach began with feature selection from a biologically meaningful gene pool—namely, genes known to interact with dioxins—followed by stepwise logistic regression and refinement using a RF algorithm. This combined strategy takes full advantage of ensemble learning. RF are particularly well-suited for high-dimensional genomic data, as they accommodate situations where the number of predictors exceeds the number of samples, perform internal feature subsampling for robust selection, capture nonlinear gene-gene interactions, and reduce overfitting compared with single decision trees [33]. Ultimately, our eight-gene model is more concise, mechanistically grounded, and better suited for clinical translation than previously proposed blood-based diagnostic tools.

Our 16-gene prognostic model constructed using the RSF algorithm demonstrated superior performance compared with several recently reported signatures and offered additional clinically meaningful insights. RSF combines multiple decision trees to capture complex and non-linear relationships between gene expression and survival outcomes. Unlike traditional parametric approaches, it is data-adaptive and does not require proportional hazards assumptions or prespecified distributional forms [34]. This property makes RSF particularly suitable for high-dimensional genomic data [34]. In the present study, cross-cohort validation confirmed that the RSF-derived risk score consistently ranked among the top prognostic signatures and retained stable predictive power across multiple external datasets. Importantly, the risk score exhibited a positive association with TMB and stratified patients into subgroups with distinct survival outcomes under immune checkpoint blockade. High TMB has been recognised as a predictive biomarker for immunotherapy response in large-scale clinical trials such as CheckMate 227 and KEYNOTE-158 [35], and the RSF signature may therefore reflect genomic instability and neoantigen load that underlie treatment efficacy. In addition, drug-sensitivity analyses indicated that patients stratified by the RSF riskscore differed in predicted responses to commonly used chemotherapeutic agents, suggesting potential clinical utility for guiding therapeutic strategies and optimizing treatment combinations.

SLC15A2 is a potential core gene identified in our study for LUAD, encoding the proton-coupled peptide transporter PEPT2, which is widely expressed and prominently localized in the lung epithelium. In human lung, PEPT2 mRNA/protein are detected in bronchial epithelial cells and alveolar type II pneumocytes, consistent with a role in trans-epithelial handling of di-/tripeptides and peptide-like drugs [36]. This physiological positioning suggests that changes in SLC15A2 could influence nutrient shuttling, epithelial stress responses, and local pharmacokinetics within the pulmonary microenvironment. Cancer-relevant data indicate that SLC15A2 may modulate tumour behaviour and treatment response. In hepatocellular carcinoma, germline variation in SLC15A2 was associated with sorafenib benefit, and cell models carrying the variant allele exhibited greater drug sensitivity, implicating PEPT2-mediated transport in therapeutic efficacy [37]. Taken together, the pulmonary distribution of PEPT2 and its emerging links to anticancer drug response support SLC15A2 as a biologically coherent candidate in lung cancer research.

Despite the important findings of this study, several limitations should be acknowledged. First, although multiple public datasets and independent validation cohorts were included, this study was mainly based on retrospective transcriptomic data from public repositories. Potential dataset bias, platform heterogeneity, batch effects, and population-specific differences cannot be completely excluded. Moreover, although algorithm-specific cross-validation and external validation were incorporated into the machine-learning workflow, a strict nested cross-validation framework was not implemented; therefore, residual overfitting, particularly in the diagnostic model, may still exist. Second, the mechanistic role of SLC15A2 requires further validation. Although SLC15A2 overexpression suppressed LUAD cell proliferation and invasion, and an in silico virtual-knockout analysis provided additional computational evidence for its potential downstream regulatory pathways, virtual knockout cannot replace wet-laboratory loss-of-function experiments. Future studies should include siRNA/shRNA-mediated knockdown, CRISPR-based knockout, rescue experiments, and in vivo assays to confirm the causal role of SLC15A2 in LUAD. Third, this study mainly relied on transcriptomic data, without integration of proteomics, epigenomics, metabolomics, or spatial transcriptomics. Multi-omics analyses may provide a more comprehensive understanding of SLC15A2-mediated regulation in dioxin-related LUAD progression. Finally, the clinical applicability of the diagnostic and prognostic models remains preliminary and should be further evaluated in large-scale, prospective, multicenter cohorts with standardized sample processing and clinically relevant endpoints.

## Conclusion

In this study, diagnostic and prognostic models for LUAD were successfully established using transcriptomic data, demonstrating robust predictive performance. Among the identified hub genes, SLC15A2 was consistently incorporated into both models and experimentally validated as downregulated in LUAD. Functional assays further indicated its tumor-suppressive role, suggesting that SLC15A2 may serve not only as a biomarker for diagnosis and prognosis but also as a potential tumor suppressor gene in LUAD.

## Electronic Supplementary Material

Below is the link to the electronic supplementary material.

Supplementary Material 1

Supplementary Material 2

Supplementary Material 3

Supplementary Material 4

Supplementary Material 5

Supplementary Material 6

Supplementary Material 7

Supplementary Material 8

## Data Availability

No datasets were generated or analysed during the current study.
