# Supplementary material for: Integrative analysis and experimental validation of dioxin-interacting genes reveal diagnostic and prognostic biomarkers in lung adenocarcinoma
Source: Clin Exp Med. 2026 May 26;26(1):277. doi: 10.1007/s10238-026-02187-3 (PMC13391747; doi:10.1007/s10238-026-02187-3)
Supplement: Supplementary file 1 — Supplementary Material 1 [file 10238_2026_2187_MOESM1_ESM.doc]

**Supplementary Figure 1.** Comparison of the performance of the proposed prognostic model with previously published models.


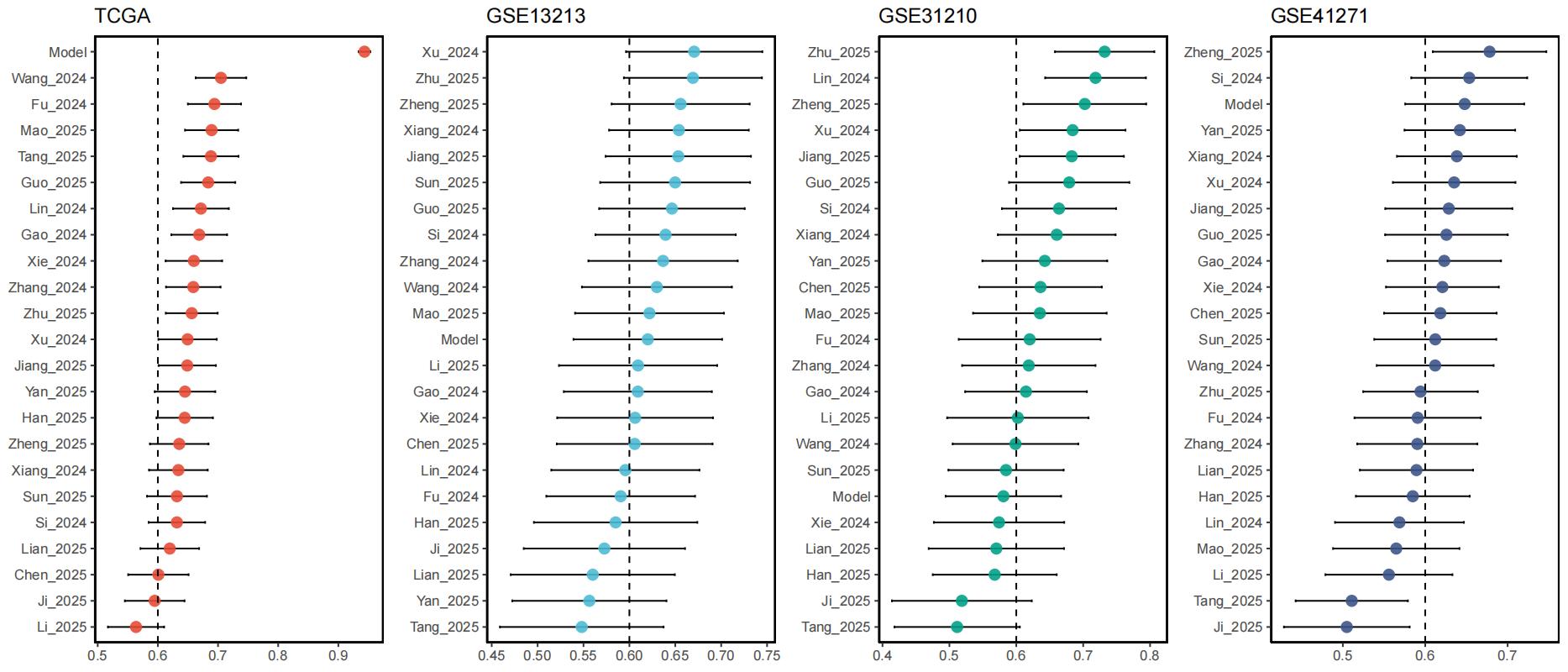


1 Wang J, Zhang H, Feng Y, Gong X, Song X, Wei M, Hu Y, Li J. Aging-Related Gene-Based Prognostic Model for Lung Adenocarcinoma: Insights into Tumor Microenvironment and Therapeutic Implications. Int J Mol Sci 2024 10.3390/ijms25135372

2 Fu D, Zhang B, Fan W, Zeng F, Feng J, Wang X. Fatty acid metabolism prognostic signature predicts tumor immune microenvironment and immunotherapy, and identifies tumorigenic role of MOGAT2 in lung adenocarcinoma. Front Immunol 2024 10.3389/fimmu.2024.1456719

3 Mao J, Li L, Sun H, Han J, Li J, Dong CS, Zhao H. Investigation of sphingolipid-related genes in lung adenocarcinoma. Front Mol Biosci 2025 10.3389/fmolb.2025.1548655

4 Tang H, Zhu J, Wang Y, Zhang J, Zhou J, Chen Z. Defining lung adenocarcinoma subtypes with glucocorticoid-related genes and constructing a prognostic index for immunotherapy guidance. J Thorac Dis 2025 10.21037/jtd-25-380

5 Yan F, Guo Q, Zheng R, Ying J. Predictive performance of a centrosome-associated prognostic model in prognosis and immunotherapy of lung adenocarcinoma. Anal Biochem 2025 10.1016/j.ab.2024.115731

6 Lin H, Yin W. Telomere-related prognostic signature for survival assessments in lung adenocarcinoma. Transl Cancer Res 2024 10.21037/tcr-24-552

7 Gao S, Huang J, Zhao R, He H, Zhang J, Wen X. Comprehensive analysis of multiple regulated cell death risk signatures in lung adenocarcinoma. Heliyon 2024 10.1016/j.heliyon.2024.e38641

8 Xie Y, Chen H, Zhang X, Zhang J, Zhang K, Wang X, Min S, Wang X, Lian C. Integration of the bulk transcriptome and single-cell transcriptome reveals efferocytosis features in lung adenocarcinoma prognosis and immunotherapy by combining deep learning. Cancer Cell Int 2024 10.1186/s12935-024-0388-1

9 Zhang Z, Zhang P, Xie J, Cui Y, Wang S, Yue D. Five-gene prognostic model based on autophagy-dependent cell death for predicting prognosis in lung adenocarcinoma. Sci Rep 2024 10.1038/s41598-024-26449-9

10 Zhu M, Wang L, Chen F. Harnessing natural killer cell-related genes for prognostic and therapeutic advances in lung adenocarcinoma: a predictive model for survival and immunotherapy outcomes. Transl Cancer Res 2025 10.21037/tcr-2025-380

11 Xu B, Zhang L, Lin L, Lin Y, Lai F. Development of a novel disulfidptosis-correlated m6A/m1A/m5C/m7G gene signature to predict prognosis and therapeutic response for lung adenocarcinoma patients by integrated machine-learning. Horm Cancer 2024 10.1007/s12672-024-01532-9

12 Jiang W, Zhang F, Tang Z, Xu S, Zhang Y, Liu L, Zhong D, Liu Y. Prediction of prognosis and immune response in lung adenocarcinoma based on mitophagy and lactate-related gene signatures. Int J Clin Oncol 2025 10.1007/s10147-024-02479-3

13 Han P, Guda C, Liu Q. An efficient epithelial-mesenchymal transition-related gene signature for predicting the survival of patients with lung adenocarcinoma. Transl Cancer Res 2025 10.21037/tcr-2025-1455

14 Zheng W, Zhou C, Xue Z, Qiao L, Wang J, Lu F. Integrative analysis of a novel signature incorporating metabolism and stemness-related genes for risk stratification and assessing clinical outcomes and therapeutic responses in lung adenocarcinoma. BMC Cancer 2025 10.1186/s12885-025-1591-7

15 Xiang H, Kasajima R, Azuma K, Tagami T, Hagiwara A, Nakahara Y, Saito H, Igarashi Y, Wei F, Ban T, Yoshihara M, Nakamura Y, Sato S, Koizume S, Tamura T, Sasada T, Miyagi Y. Multi-omics analysis-based clinical and functional significance of a novel prognostic and immunotherapeutic gene signature derived from amino acid metabolism pathways in lung adenocarcinoma. Front Immunol 2024 10.3389/fimmu.2024.1361992

16 Sun D, Duan X, Li N, Qiao O, Hou Y, Ma Z, Liu S, Gong Y, Liu Z. Construction of ubiquitination-related risk model for predicting prognosis in lung adenocarcinoma. Sci Rep 2025 10.1038/s41598-025-11787-2

17 Si Y, Zhao Z, Meng X, Zhao K. RNA-seq and bulk RNA-seq data analysis of cancer-associated fibroblasts (CAF) in LUAD to construct a CAF-based risk signature. Sci Rep 2024 10.1038/s41598-024-23243-8

18 Lian LZ, Huang F, Lang J, Yuan JF, Hu PP. Metabolic Reprogramming-Related Genes in Lung Adenocarcinoma: Identification and Prognostic Model Construction. World J Oncol 2025 10.14740/wjon2604

19 Chen J, Huang J, Shen L. Construction of lung adenocarcinoma subtype and prognosis model based on fatty acid metabolism-related genes. Horm Cancer 2025 10.1007/s12672-025-01608-y

20 Ji T, Jiang J, Wang X, Yang K, Wang S, Pan B. Single-cell transcriptomics and machine learning unveil ferroptosis features in tumor-associated macrophages: Prognostic model and therapeutic strategies for lung adenocarcinoma. Front Pharmacol 2025 10.3389/fphar.2025.1598756

21 Li H, Li G, Gao X, Chen C, Cui Z, Cao X, Su J. Development of a reliable risk prognostic model for lung adenocarcinoma based on the genes related to endotheliocyte senescence. Sci Rep 2025 10.1038/s41598-025-12604-8
