# Supplementary material for: Integrative analysis and experimental validation of dioxin-interacting genes reveal diagnostic and prognostic biomarkers in lung adenocarcinoma
Source: Clin Exp Med. 2026 May 26;26(1):277. doi: 10.1007/s10238-026-02187-3 (PMC13391747; doi:10.1007/s10238-026-02187-3)

**Supplementary Figure 2.** Overview of single-cell RNA sequencing data quality assessment across all samples. The violin plots summarize four essential metrics measured prior to data filtering: nFeature_RNA, indicating the diversity of transcripts captured per cell; nCount_RNA, representing the total UMI counts and thus the sequencing depth; percent.mt, reflecting the proportion of mitochondrial transcripts, with elevated values often associated with stressed or dying cells; and percent.rb, showing the relative abundance of ribosomal RNA expression. Together, these parameters provide an overall evaluation of dataset quality and guide the exclusion of low-quality cells before downstream analyses.


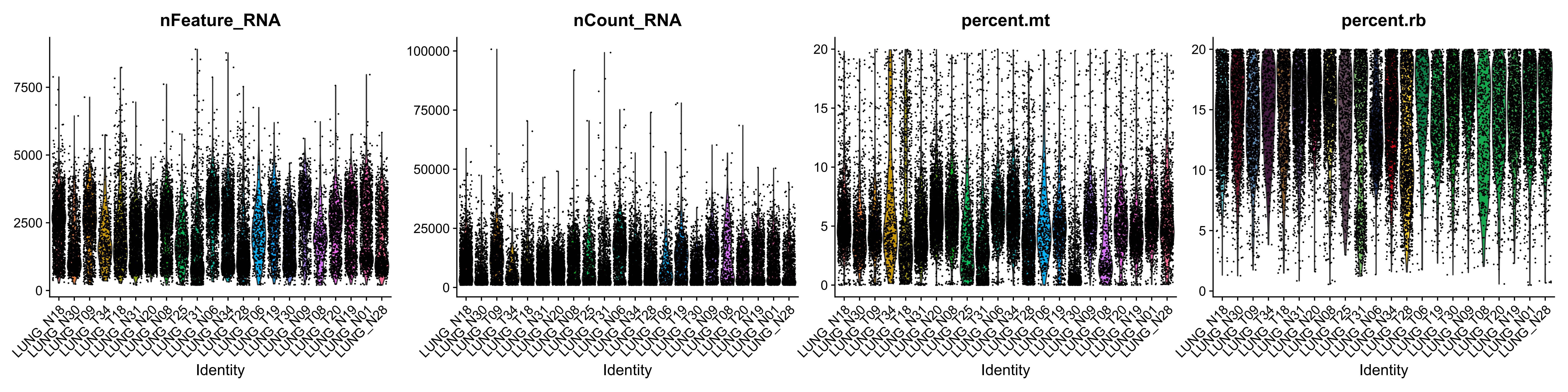

Supplement: Supplementary file 2 — Supplementary Material 2 [file 10238_2026_2187_MOESM2_ESM.doc]
