# Supplementary material for: Integrative analysis and experimental validation of dioxin-interacting genes reveal diagnostic and prognostic biomarkers in lung adenocarcinoma
Source: Clin Exp Med. 2026 May 26;26(1):277. doi: 10.1007/s10238-026-02187-3 (PMC13391747; doi:10.1007/s10238-026-02187-3)
Supplement: Supplementary file 3 — Supplementary Material 3 [file 10238_2026_2187_MOESM3_ESM.doc]

Supplementary Table 1. The characteristics of the public datasets.

| Datasets | Platform | No. of included LUAD patients | No. Of Control | Country |
| --- | --- | --- | --- | --- |
| TCGA | Illumina Hiseq | 478 | 59 | USA |
| GSE13213 | GPL6480 | 117 | 0 | Japan |
| GSE31210 | GPL570 | 226 | 0 | Japan |
| GSE41271 | GPL6884 | 181 | 0 | USA |
| GSE135304 | GPL10558 | 244 | 308 | USA |
| GSE20189 | GPL571 | 73 | 80 | USA |
| GSE131907 | GPL16791 | 11 | 11 | South Korea |
| GSE135222 | GPL16791 | 27 (NSCLC) | 0 | South Korea |
