# Supplementary material for: Integrative analysis and experimental validation of dioxin-interacting genes reveal diagnostic and prognostic biomarkers in lung adenocarcinoma
Source: Clin Exp Med. 2026 May 26;26(1):277. doi: 10.1007/s10238-026-02187-3 (PMC13391747; doi:10.1007/s10238-026-02187-3)
Supplement: Supplementary file 4 — Supplementary Material 4 [file 10238_2026_2187_MOESM4_ESM.doc]

**Supplementary Table 2.** Intersection of differentially expressed genes (DEGs) with genes from the brown module, resulting in a total of 170 genes.

| MGLL |
| --- |
| ST3GAL5 |
| MAP2K6 |
| GLDC |
| RNASE1 |
| EPHA4 |
| ABCC3 |
| PFN2 |
| EGR1 |
| AUTS2 |
| PHGDH |
| SLC1A7 |
| SFTPD |
| GCLC |
| ALPL |
| CEACAM6 |
| PDZK1IP1 |
| TNNT1 |
| CDC20 |
| UBE2C |
| ASF1B |
| CEP55 |
| TK1 |
| TPX2 |
| MELK |
| RRM2 |
| KPNA2 |
| CDCA8 |
| AURKB |
| CCNB2 |
| PRR11 |
| CDKN3 |
| TOP2A |
| CCNA2 |
| GTSE1 |
| NCAPG |
| CACNA2D2 |
| MCM6 |
| KIF23 |
| TTK |
| TRIP13 |
| NDC80 |
| NUSAP1 |
| CHEK1 |
| BUB1 |
| MAD2L1 |
| ZWINT |
| SPAG5 |
| MCM4 |
| CDT1 |
| KIF11 |
| PRC1 |
| PLK4 |
| CENPM |
| MCM2 |
| AFF3 |
| RAD51AP1 |
| GINS2 |
| CCNE1 |
| DTL |
| EIF4EBP1 |
| TYMS |
| PLOD2 |
| SELENBP1 |
| CENPE |
| PTTG1 |
| SLC2A1 |
| BRCA1 |
| MMD |
| AURKA |
| ASPM |
| KIF14 |
| ZBTB16 |
| E2F1 |
| PMAIP1 |
| VIPR1 |
| SORBS2 |
| MAOA |
| RPL39L |
| ABCA3 |
| ELOVL6 |
| CTSH |
| FOS |
| EGLN3 |
| COCH |
| LPL |
| FMO5 |
| AQP3 |
| ORM1 |
| RACGAP1 |
| FEN1 |
| RFC4 |
| NFIX |
| PSRC1 |
| CRY2 |
| MTHFD2 |
| TACC3 |
| CENPN |
| TRAIP |
| CKS1B |
| RNASEH2A |
| NR3C2 |
| POLE2 |
| CKS2 |
| GAPDH |
| NEDD9 |
| BLM |
| TLR5 |
| POLQ |
| TIMELESS |
| LMNB1 |
| PCNA |
| NME1 |
| CITED2 |
| CISH |
| EZH2 |
| TEF |
| E2F2 |
| GINS4 |
| MCM7 |
| RPS6KA2 |
| CDCA4 |
| DAPK2 |
| STIL |
| TMPO |
| GPD1L |
| IL6R |
| NOD1 |
| RFTN1 |
| KLF2 |
| STMN1 |
| HMGB2 |
| CDC7 |
| ATAD2 |
| SLC15A2 |
| AK1 |
| PRIM1 |
| NCAPD2 |
| KNTC1 |
| DUSP1 |
| SLC7A5 |
| PSAT1 |
| PTPRE |
| SKP2 |
| GGH |
| ASNS |
| PXMP4 |
| ZFP36 |
| ACSL5 |
| CYB5A |
| TRIB3 |
| PGM5 |
| IGF2BP3 |
| KCNJ15 |
| TNNC1 |
| CLIC3 |
| ACSM3 |
| CFI |
| PFKP |
| STEAP4 |
| MLLT11 |
| MUC1 |
| TXNRD1 |
| LYPD3 |
| ADRA2A |
| SUSD4 |
| ADM |
| SLC4A4 |
| HOXA10 |
| QPCT |
