# Supplementary material for: Integrative analysis and experimental validation of dioxin-interacting genes reveal diagnostic and prognostic biomarkers in lung adenocarcinoma
Source: Clin Exp Med. 2026 May 26;26(1):277. doi: 10.1007/s10238-026-02187-3 (PMC13391747; doi:10.1007/s10238-026-02187-3)
Supplement: Supplementary file 5 — Supplementary Material 5 [file 10238_2026_2187_MOESM5_ESM.doc]

**Supplementary Table 3.** Features used for the construction of the diagnostic model.

| CITED2 |
| --- |
| ADRA2A |
| NEDD9 |
| SLC15A2 |
| SLC1A7 |
| PDZK1IP1 |
| MTHFD2 |
| ZBTB16 |
