# Supplementary material for: Integrative analysis and experimental validation of dioxin-interacting genes reveal diagnostic and prognostic biomarkers in lung adenocarcinoma
Source: Clin Exp Med. 2026 May 26;26(1):277. doi: 10.1007/s10238-026-02187-3 (PMC13391747; doi:10.1007/s10238-026-02187-3)
Supplement: Supplementary file 6 — Supplementary Material 6 [file 10238_2026_2187_MOESM6_ESM.doc]

**Supplementary Table 4.** Features used for the construction of the prognostic model.

| SLC2A1 |
| --- |
| GAPDH |
| SLC7A5 |
| CEACAM6 |
| SFTPD |
| SUSD4 |
| LYPD3 |
| PTTG1 |
| SKP2 |
| PFKP |
| DAPK2 |
| TNNT1 |
| ABCA3 |
| TXNRD1 |
| SLC15A2 |
