# Supplementary material for: Integrative analysis and experimental validation of dioxin-interacting genes reveal diagnostic and prognostic biomarkers in lung adenocarcinoma
Source: Clin Exp Med. 2026 May 26;26(1):277. doi: 10.1007/s10238-026-02187-3 (PMC13391747; doi:10.1007/s10238-026-02187-3)
Supplement: Supplementary file 7 — Supplementary Material 7 [file 10238_2026_2187_MOESM7_ESM.doc]

Supplementary Table 5. Association of risk scores with somatic variant.

| **gene** | **H-wild** | **H-mutation** | **L-wild** | **L-mutation** | **P value** |
| --- | --- | --- | --- | --- | --- |
| TP53 | 156(47.42%) | 173(52.58%) | 99(71.22%) | 40(28.78%) | 3.76182064073467e-06 |
| TTN | 164(49.85%) | 165(50.15%) | 95(68.35%) | 44(31.65%) | 0.000348529933016975 |
| CSMD3 | 188(57.14%) | 141(42.86%) | 103(74.1%) | 36(25.9%) | 0.000801047807260731 |
| COL22A1 | 289(87.84%) | 40(12.16%) | 136(97.84%) | 3(2.16%) | 0.00116639891172499 |
| SI | 269(81.76%) | 60(18.24%) | 130(93.53%) | 9(6.47%) | 0.00170780996178278 |
| HCN1 | 286(86.93%) | 43(13.07%) | 134(96.4%) | 5(3.6%) | 0.0035032286318067 |
| LCT | 298(90.58%) | 31(9.42%) | 137(98.56%) | 2(1.44%) | 0.0039128863131971 |
| GABRA4 | 303(92.1%) | 26(7.9%) | 138(99.28%) | 1(0.72%) | 0.00467643933787449 |
| PCDH15 | 258(78.42%) | 71(21.58%) | 125(89.93%) | 14(10.07%) | 0.00480807630169814 |
| NTRK3 | 295(89.67%) | 34(10.33%) | 136(97.84%) | 3(2.16%) | 0.00498812621464256 |
| HRNR | 284(86.32%) | 45(13.68%) | 133(95.68%) | 6(4.32%) | 0.00499498286625001 |
| NPAP1 | 268(81.46%) | 61(18.54%) | 128(92.09%) | 11(7.91%) | 0.00558047081173877 |
| SPTA1 | 244(74.16%) | 85(25.84%) | 120(86.33%) | 19(13.67%) | 0.00558396535988231 |
| CARD11 | 304(92.4%) | 25(7.6%) | 138(99.28%) | 1(0.72%) | 0.00599664765558586 |
| SLC39A12 | 296(89.97%) | 33(10.03%) | 136(97.84%) | 3(2.16%) | 0.00632426944435653 |
| GRM7 | 300(91.19%) | 29(8.81%) | 137(98.56%) | 2(1.44%) | 0.00636674960677162 |
| ITPRID1 | 300(91.19%) | 29(8.81%) | 137(98.56%) | 2(1.44%) | 0.00636674960677162 |
| KCNU1 | 300(91.19%) | 29(8.81%) | 137(98.56%) | 2(1.44%) | 0.00636674960677162 |
| TRPC4 | 300(91.19%) | 29(8.81%) | 137(98.56%) | 2(1.44%) | 0.00636674960677162 |
| BSN | 306(93.01%) | 23(6.99%) | 138(99.28%) | 1(0.72%) | 0.00984328006048991 |
| AMER3 | 302(91.79%) | 27(8.21%) | 137(98.56%) | 2(1.44%) | 0.0103146291653634 |
| FAT1 | 291(88.45%) | 38(11.55%) | 134(96.4%) | 5(3.6%) | 0.0108796136170092 |
| KCNB2 | 295(89.67%) | 34(10.33%) | 135(97.12%) | 4(2.88%) | 0.0119553203276382 |
| TMPRSS15 | 295(89.67%) | 34(10.33%) | 135(97.12%) | 4(2.88%) | 0.0119553203276382 |
| OR5L1 | 299(90.88%) | 30(9.12%) | 136(97.84%) | 3(2.16%) | 0.0127760809242311 |
| CD163L1 | 308(93.62%) | 21(6.38%) | 138(99.28%) | 1(0.72%) | 0.0161237475839853 |
| NID2 | 304(92.4%) | 25(7.6%) | 137(98.56%) | 2(1.44%) | 0.0166365472028002 |
| MUC16 | 185(56.23%) | 144(43.77%) | 95(68.35%) | 44(31.65%) | 0.0193085041569105 |
| SYNE1 | 287(87.23%) | 42(12.77%) | 132(94.96%) | 7(5.04%) | 0.0197772066924836 |
| COL6A3 | 284(86.32%) | 45(13.68%) | 131(94.24%) | 8(5.76%) | 0.0207954865334747 |
| ANKRD30A | 284(86.32%) | 45(13.68%) | 131(94.24%) | 8(5.76%) | 0.0207954865334747 |
| GLI2 | 305(92.71%) | 24(7.29%) | 137(98.56%) | 2(1.44%) | 0.0210923417556926 |
| ADGRB3 | 285(86.63%) | 44(13.37%) | 131(94.24%) | 8(5.76%) | 0.0253919932284989 |
| MYCBP2 | 302(91.79%) | 27(8.21%) | 136(97.84%) | 3(2.16%) | 0.0254490978798456 |
| LRFN5 | 295(89.67%) | 34(10.33%) | 134(96.4%) | 5(3.6%) | 0.0259733707038989 |
| DYNC2H1 | 306(93.01%) | 23(6.99%) | 137(98.56%) | 2(1.44%) | 0.0267100683050344 |
| UBR4 | 299(90.88%) | 30(9.12%) | 135(97.12%) | 4(2.88%) | 0.0291170239320239 |
| TRRAP | 299(90.88%) | 30(9.12%) | 135(97.12%) | 4(2.88%) | 0.0291170239320239 |
| MYH6 | 299(90.88%) | 30(9.12%) | 135(97.12%) | 4(2.88%) | 0.0291170239320239 |
| CPS1 | 286(86.93%) | 43(13.07%) | 131(94.24%) | 8(5.76%) | 0.0309227971448555 |
| OR2T33 | 303(92.1%) | 26(7.9%) | 136(97.84%) | 3(2.16%) | 0.0319126948868981 |
| PTPRD | 268(81.46%) | 61(18.54%) | 125(89.93%) | 14(10.07%) | 0.0320139253959002 |
| COL19A1 | 296(89.97%) | 33(10.03%) | 134(96.4%) | 5(3.6%) | 0.032105950907718 |
| MYH4 | 296(89.97%) | 33(10.03%) | 134(96.4%) | 5(3.6%) | 0.032105950907718 |
| SCN3A | 300(91.19%) | 29(8.81%) | 135(97.12%) | 4(2.88%) | 0.036188698744911 |
| LRRC7 | 275(83.59%) | 54(16.41%) | 127(91.37%) | 12(8.63%) | 0.0389799200397945 |
| FAT4 | 275(83.59%) | 54(16.41%) | 127(91.37%) | 12(8.63%) | 0.0389799200397945 |
| MGA | 297(90.27%) | 32(9.73%) | 134(96.4%) | 5(3.6%) | 0.0395915826142484 |
| GRM5 | 297(90.27%) | 32(9.73%) | 134(96.4%) | 5(3.6%) | 0.0395915826142484 |
| PIK3CG | 304(92.4%) | 25(7.6%) | 136(97.84%) | 3(2.16%) | 0.0399456265720068 |
| ADAMTS12 | 261(79.33%) | 68(20.67%) | 122(87.77%) | 17(12.23%) | 0.0421096031429896 |
| OR8H2 | 308(93.62%) | 21(6.38%) | 137(98.56%) | 2(1.44%) | 0.0426749893847936 |
| NFASC | 308(93.62%) | 21(6.38%) | 137(98.56%) | 2(1.44%) | 0.0426749893847936 |
| TIAM1 | 291(88.45%) | 38(11.55%) | 132(94.96%) | 7(5.04%) | 0.0441440338701216 |
| CLCN1 | 301(91.49%) | 28(8.51%) | 135(97.12%) | 4(2.88%) | 0.0448782771681573 |
| PRKDC | 301(91.49%) | 28(8.51%) | 135(97.12%) | 4(2.88%) | 0.0448782771681573 |
| ERICH3 | 273(82.98%) | 56(17.02%) | 126(90.65%) | 13(9.35%) | 0.0459879449024979 |
| EPHA5 | 285(86.63%) | 44(13.37%) | 130(93.53%) | 9(6.47%) | 0.0463218470397331 |
| FAM135B | 276(83.89%) | 53(16.11%) | 127(91.37%) | 12(8.63%) | 0.0465078300553106 |
| HERC2 | 282(85.71%) | 47(14.29%) | 129(92.81%) | 10(7.19%) | 0.0467281802232842 |
| BOD1L1 | 298(90.58%) | 31(9.42%) | 134(96.4%) | 5(3.6%) | 0.0487009848555242 |
| APOB | 262(79.64%) | 67(20.36%) | 122(87.77%) | 17(12.23%) | 0.0495834865037408 |
| SEZ6L | 305(92.71%) | 24(7.29%) | 136(97.84%) | 3(2.16%) | 0.049905796537921 |
| MYH3 | 305(92.71%) | 24(7.29%) | 136(97.84%) | 3(2.16%) | 0.049905796537921 |
| DNAH2 | 305(92.71%) | 24(7.29%) | 136(97.84%) | 3(2.16%) | 0.049905796537921 |
| ANK3 | 305(92.71%) | 24(7.29%) | 136(97.84%) | 3(2.16%) | 0.049905796537921 |
| MAGI2 | 305(92.71%) | 24(7.29%) | 136(97.84%) | 3(2.16%) | 0.049905796537921 |
| ATP10B | 305(92.71%) | 24(7.29%) | 136(97.84%) | 3(2.16%) | 0.049905796537921 |
