# Supplementary material for: Integrative analysis and experimental validation of dioxin-interacting genes reveal diagnostic and prognostic biomarkers in lung adenocarcinoma
Source: Clin Exp Med. 2026 May 26;26(1):277. doi: 10.1007/s10238-026-02187-3 (PMC13391747; doi:10.1007/s10238-026-02187-3)
Supplement: Supplementary file 8 — Supplementary Material 8 [file 10238_2026_2187_MOESM8_ESM.doc]

Supplementary Figure 3. In silico virtual-knockout analysis of SLC15A2 in LUAD tumor-derived cells.

**(A)** Pie chart showing the proportion of significantly perturbed genes after SLC15A2 virtual knockout. Significant genes were defined as those with adjusted P values < 0.05.
**(B)** Bar plot showing the top 20 differentially regulated genes ranked by fold change after SLC15A2 virtual knockout.
**(C)** Volcano plot showing the distribution of differentially regulated genes. Red points indicate significantly perturbed genes, whereas gray points indicate non-significant genes.
**(D)** GO enrichment analysis of significantly perturbed genes.
**(E)** Pathway enrichment analysis of significantly perturbed genes. Dot size represents the number of enriched genes, and color indicates enrichment significance.
